# The Impact of Evidence-Based Transformation on Healthcare Practices at a Teaching Hospital

**DOI:** 10.7759/cureus.11744

**Published:** 2020-11-28

**Authors:** Aisha Wali, Annum Ishtiaq, Anum Rahim, Sundus Iftikhar

**Affiliations:** 1 Obstetrics and Gynecology, The Indus Hospital, Karachi, PAK; 2 Family Medicine, The Indus Hospital, Karachi, PAK; 3 Epidemiology and Public Health, The Indus Hospital, Karachi, PAK; 4 Statistics, Indus Hospital Research Center, The Indus Hospital, Karachi, PAK

**Keywords:** standard protocols, quality care, antimicrobial therapy, anemia in pregnancy, surgical site infection, average length of hospital stay, cost of antibiotics in surgical procedures

## Abstract

Objective

In this study, we aimed to compare the frequency of anemia, blood transfusions, and the use of antimicrobial therapy (AMT) before the implementation of standard protocols in obstetrics and gynecology with the data after one year of implementation at a teaching hospital.

Methods

In this retrospective observational study, the pre-intervention data (group A) were compared to the data after the implementation of standard protocols (group B). Data were retrieved from hospital electronic medical records and were entered and analyzed on SPSS Statistics version 24 (IBM, Armonk, NY).

Results

In obstetrics (n=829), anemia was observed in 43.1% vs. 26.8% (p<0.001) and transfusion rate in 5.4% vs. 0.6% (p<0.001) in the groups A and B respectively. In vaginal deliveries (VD), the use of AMT for >24 hours was 98% in group A vs. 9% in group B, and in cesarean deliveries (CD), it was 100% in group A vs. 54.5% in group B (p<0.001). The cost of AMT decreased by 78.4% in VD and by 51.1% in CD.

In gynecology (n=221), the prevalence of anemia was 22.6% in group A vs. 17.9% in group B (p=NS). In minor procedures, the use of AMT for >24 hours was 76.7% vs. 8.4% (p<0.001), and in major procedures, it was 86.5% vs. 38% (p<0.001) between the two groups. The cost of AMT decreased by 79.5% in minor procedures and 26.4% in major procedures.

Conclusion

The implementation of quality standards can bring about significant improvements in clinical outcomes in a short period of time.

## Introduction

Poor patient outcomes are largely caused by substandard medical care, particularly in low- and middle-income countries (LMICs) where resources are often insufficient and in short supply. Data regarding the frequency, intensity, and types of such practices are scarce in Southeast Asia, while they have been widely studied in the rest of the world. Nevertheless, patient safety and healthcare quality must be understood in the context of various cultural, political, and social contexts in order to develop relevant intervention strategies [1]. High maternal and infant mortality rates in Southeast Asia highlight the seriousness of the issue [2].

Pakistan has an extensive physical healthcare infrastructure, but most of the centers in remote areas of the country are not functional. Therefore, the successful delivery of quality care to its people has not fully materialized [3]. Improving the quality of healthcare involves sound local approaches to achieve the best possible outcomes [4]. A top-down approach involves the formulation of standard guidelines, and the concurrent bottom-up attempt includes ensuring compliance with these protocols. Cost and efficiency are the most important qualitative attributes to evaluate healthcare services. Maximizing resources and skills without quality optimization does not produce the desired results. This change from volume- to value-based care includes a re-examination of the existing traditional management protocols by means of an evidence-based lens, with greater emphasis on best practices [3,5]. Information management systems are also important to healthcare and can improve quality and performance apart from bringing down costs.

In October 2015, The Indus Hospital (TIH) took over the administration of an existing maternity hospital to establish its department of obstetrics and gynecology. The challenges included a high number of unregistered women and hence a high frequency of anemia leading to the liberal use of blood transfusions. Another challenge was the injudicious use of antimicrobial therapy (AMT). The overuse of antimicrobials not only exponentiates the financial burden on the healthcare system and prolongs hospital stay but also, in parallel, compromises the general health and immunity of the human body and results in the emergence of resistant strains [6].

Quality is the foundation of healthcare in the modern world; developing countries are also realizing its importance and are therefore adopting standard management protocols in their healthcare systems [6-7]. Our department of obstetrics and gynecology developed departmental protocols in accordance with the international standards and implemented them stepwise from the third quarter of 2016 onwards. These included guidelines for antenatal care with a policy to register women for childbirth by 20 weeks of gestation [8], protocols for treating anemia in pregnancy [9], recommendations for blood transfusions [10], and guidelines for the appropriate use of AMT [11-12].

Additionally, standardized protocols for infection control and surgical procedures were also introduced [13-14]. Health education sessions for pregnant women were also initiated as structured modules based on verbal, pictorial, and audiovisual aids. These were conducted by a team of health educators, nutritionists, and midwives.

The primary objective of this study was to compare the frequency of anemia, blood transfusions, and the use of AMT in deliveries and surgical procedures among women who used the facility during February-April 2016 with those who used it in February-April 2017 (one year after the implementation of standard care). The secondary objectives were to compare the cost of the AMT used on women undergoing procedures during these periods and to measure the difference in surgical site infections (SSI) and the average length of stay (ALOS).

## Materials and methods

A total of 1,050 women were included in this retrospective observational study that was conducted at the Sheikh Saeed Memorial campus of TIH. We sought and attained the Institutional Review Board (IRB) exemption (#IRD_IRB_2017_06_002). Electronic medical records were reviewed and data of women undergoing procedures from February to April 2016 (group A) were compared with that of women undergoing procedures from February to April 2016 (group B).

Women who delivered before 34 completed weeks and those who had antepartum hemorrhage, hemoglobinopathies, or chronic renal failure were excluded from the study. All other women undergoing obstetric procedures including vaginal or caesarian deliveries (n=829) and gynecological procedures (n=221) were included. We chose three indicators to evaluate improvement in clinical outcomes: (i) frequency of anemia - defined as the number of women with a hemoglobin level of <10.5 g/dl at the time of delivery or gynecologic procedure out of the total number of women who delivered or underwent gynecologic procedures respectively, (ii) blood transfusion rate - defined as the number of women who received blood transfusion during pregnancy or peripartum period and before, during, or after gynecologic procedures out of the total number of women who delivered or underwent gynecologic procedures respectively, and (iii) the use of AMT. To compare the cost of AMT, the fixed price of a particular drug used in 2016 was applied to that used in 2017. The cost of AMT was calculated by multiplying the price of a drug by the number of doses used of that particular drug.

Data were entered and analyzed on SPSS Statistics version 24 (IBM, Armonk, NY). Frequencies and percentages were determined for categorical variables whereas median and interquartile ranges were calculated for non-normal continuous variables. The Mann-Whitney U test was applied to assess the significant differences between group A (2016) and group B (2017) for quantitative variables including the difference in cost reduction of AMT. Chi-square/Fisher's exact test was performed to evaluate differences between categorical variables in both groups. A p-value of <0.05 was considered statistically significant for all analyses.

## Results

The data of 1,050 women were analyzed. Obstetrics included 829 women (group A: 354; group B: 475) and gynecology included 221 in total (group A: 67; group B: 154).

Obstetrics

**Figure 1:**
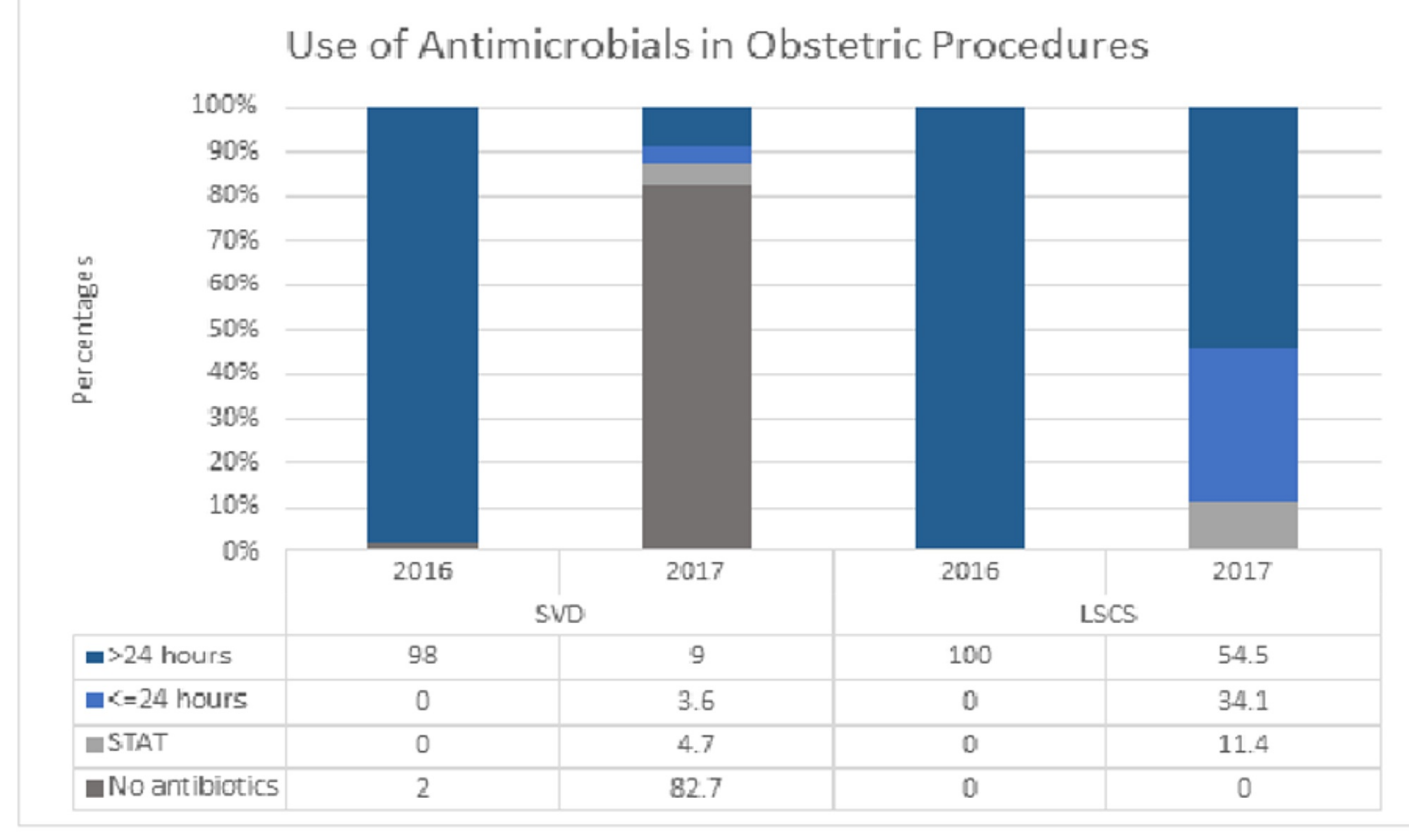
Comparison between AMT data of 2016 and 2017 for obstetric procedures AMT: antimicrobial therapy; SVD: spontaneous vaginal delivery; LSCS: lower segment cesarean section

Table 1 illustrates that the age, parity, and occupation were comparable in both the groups while significantly more women were illiterate in group A compared to group B (28% vs. 18%, p<0.001) (Table 1). The duration of antenatal care was four weeks more in group B (p<0.001). The frequency of anemia was comparable at the time of registration in both the groups (44.7% vs. 43.7%, p=NS), while at the time of delivery, it was significantly reduced in group B (43.1% vs. 26.8%, p<0.001). The transfusion rate decreased from 5.4% in group A to 0.6% in group B (p<0.001) (Table 1). The duration of AMT was categorized into the following types: (a) no antimicrobials, (b) single (STAT) dose, (c) less than 24 hours, and (d) more than 24 hours. Among women who delivered vaginally, 98% received AMT for more than 24 hours in group A, which decreased to 9% in group B. In cesarean delivery (CD), 100% of women received AMT for more than 24 hours in group A compared to 54.5% in group B (p<0.001). Overall significant reduction in the use of AMT was seen in all categories (Figure 1). The cost of AMT decreased by 78.4% in women with spontaneous vaginal delivery (SVD) and by 51.1% in women undergoing CD (Table 3). SSI in CD decreased from 7.1% in group A to 3.6% in group B. ALOS decreased by one day in both vaginal delivery (VD) and CD (p<0.001).

**Table 1:** Baseline characteristics and outcomes of obstetric patients ^Ŧ^T-test; ^¶^Mann-Whitney U test; ^§^Chi-square test; ^ƚ^Fisher's exact test; *p-value of <0.05; **p-value of <0.0001 IQR: interquartile range; Hb: hemoglobin; ALOS: average length of stay

Variables	Group A (n=354)	Group B (n=475)	P-value
Age in years, median (IQR)	26 (23-29.8)	26 (23-30)	0.888^¶^
Parity			
Primigravida, n (%)	91 (26.7)	146 (31.1)	0.333^ƚ^
Multigravida, n (%)	231 (67.7)	294 (62.7)	
Grand multigravida, n (%)	19 (5.6)	29 (6.2)	
Mode of delivery			
Spontaneous vaginal delivery, n (%)	198 (55.9)	255 (53.7)	0.063^§^
Cesarean delivery, n (%)	156 (44.0)	220 (46.3)	
Education			
Illiterate, n (%)	94 (28.3)	86 (18.2)	<0.0001**^§^
Primary, n (%)	61 (18.4)	50 (10.6)	
Matriculation and below, n (%)	141 (42.5)	252 (53.3)	
Above matriculation, n (%)	36 (10.8)	85 (18.0)	
Occupation			
Not working, n (%)	339 (95.7)	467 (98.3)	0.352^ƚ^
Gestational age at booking, weeks, median (IQR)	28 (24-31)	24 (21-27)	<0.0001**^¶^
Average antenatal care, weeks, median (IQR)	11 (8-15)	15 (12-18)	<0.0001**^¶^
Gestational age at delivery, weeks, median (IQR)	38 (37-39)	38 (37-39)	0.471^¶^
Women with pre-delivery anemia (Hb of <10.5 g/dl), n (%)	140 (43.1)	126 (26.8)	<0.0001**^§^
Women receiving blood transfusion, n (%)	19 (5.4)	3 (0.6)	<0.0001**^ƚ^
Infective morbidity			
Surgical site infection, n (%)	11 (7.1)	8 (3.6)	0.130^ƚ^
Infected episiotomy, n (%)	2 (1.0)	4 (1.6)	
Urinary tract infection, n (%)	1 (0.5)	4 (1.6)	
ALOS			
Cesarean delivery, days, median (IQR)	3 (3-4)	2 (2-3)	<0.0001**^¶^
Vagina delivery, days, median (IQR)	2 (2-2)	1 (1-1)	

Gynecology

**Figure 2:**
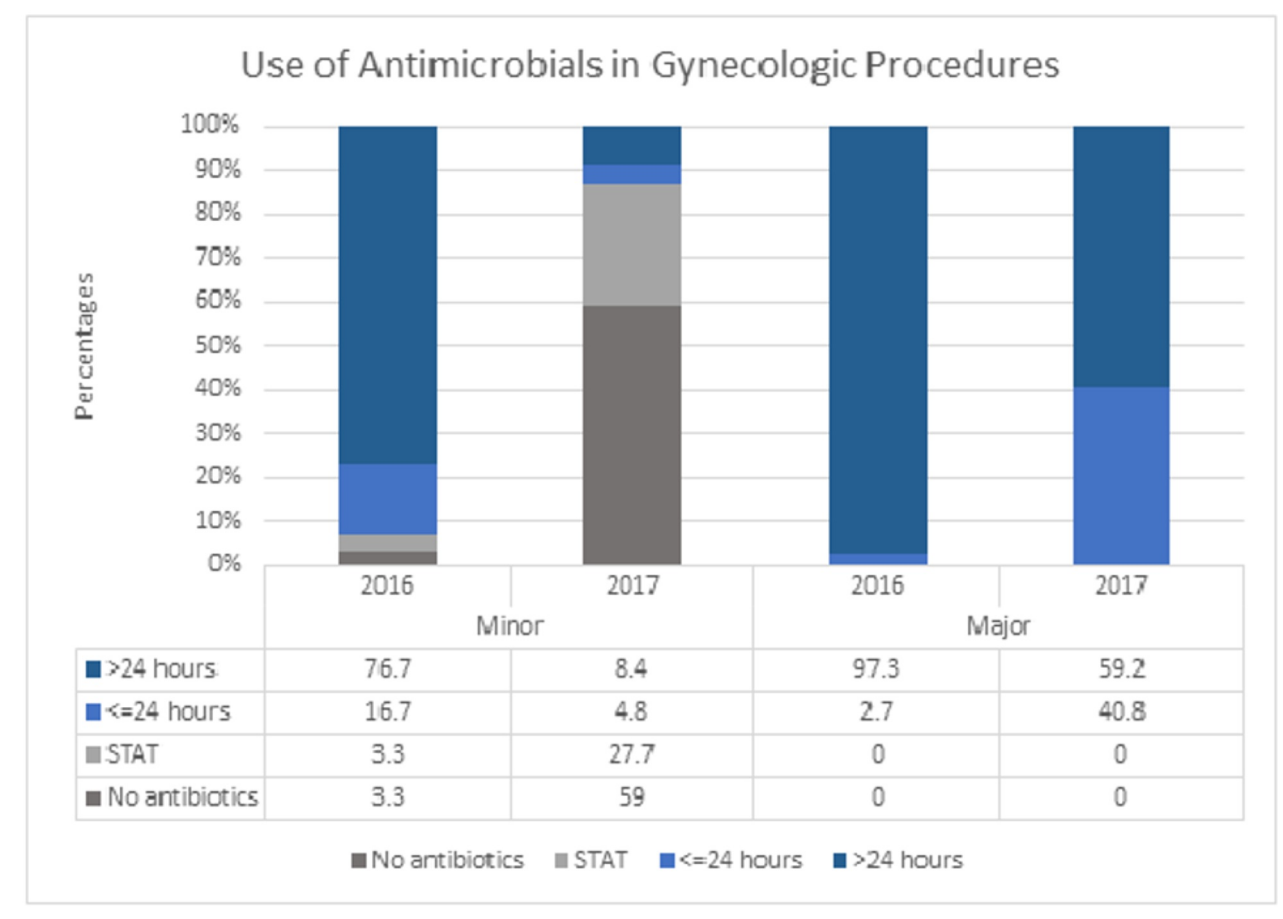
Comparison between AMT data of 2016 and 2017 for gynecologic procedures AMT: antimicrobial therapy

The baseline data were comparable in group A and group B (Table 2) except that more women were illiterate (p=0.001) and working (p=0.04) in group A. Minor emergency cases were more prevalent in group B (10% vs. 32%, p=0.001). The frequency of anemia was comparable in both the groups at registration (24.2% vs. 30.9%, p=NS), while at the time of surgery, it was 22.6% in group A compared to 17.9% in group B (p=NS). There was no significant difference in the transfusion rate between the groups (16.1% vs. 17.1%). The use of AMT was significantly reduced in all categories (Figure 2). In minor procedures, the use of AMT for more than 24 hours was 76.7% in group A compared to 8.4% in group B (p<0.001), and in major procedures, it was 97.3% in group A compared to 59.2% in group B (p<0.001). The cost of AMT was reduced by 79.5% in women with minor procedures and by 26.4% in women undergoing major procedures. SSI was significantly reduced in group B (13.5% vs. 2.8%). ALOS was reduced by one day in minor procedures and by two days in major procedures (p=0.000), while 18.3% of admissions were daycare in group B, a service that was nonexistent for women in group A (Table 3).

**Table 2:** Baseline characteristics and outcomes of gynecology patients ^Ŧ^T-test; ^¶^Mann-Whitney U test; ^§^Chi-square test; ^ƚ^Fisher's exact test; *p-value of <0.05; **p-value of <0.0001 IQR: interquartile range; Hb: hemoglobin; SSI: surgical site infections; ALOS: average length of stay

Variables	Group A (n=67)	Group B (n =154)	P-value
Age in years, median (IQR)	40 (31-46)	36 (29-43.3)	0.017*^¶^
Education			
Illiterate, n (%)	25 (41.0)	37 (26.2)	0.010*^§^
Primary, n (%)	14 (23.0)	22 (15.6)	
Matriculation and below, n (%)	12 (19.7)	62 (44.0)	
Above matriculation, n (%)	10 (16.4)	20 (14.2)	
Employment			
Not working, n (%)	58 (86.6)	147 (95.4)	0.010*^ƚ^
Comorbidities			
Diabetes mellitus, n (%)	5 (7.6)	12 (8.3)	0.402^§^
Hypertension, n (%)	11 (16.7)	15 (10.4)	
Others, n (%)	7 (10.6)	14 (9.7)	
Procedure type for minor surgeries, n (%)			
Emergency, n (%)	3 (10)	27 (32.9)	0.015*^§^
Elective, n (%)	27 (90)	55 (67.1)	
Procedure type for major surgeries			
Emergency, n (%)	1 (2.8)	3 (4.2)	1.000^ƚ^
Elective, n (%)	35 (97.2)	68 (95.8)	
Women with pre-procedure anemia (Hb of <10.5 g/dl), n (%)	14 (22.6)	22 (17.9)	0.446^§^
Blood transfusion, n (%)	10 (16.1)	22 (17.9)	0.765^§^
Infective morbidity in major procedures			
SSI, n (%)	9 (13.5)	4 (2.8)	0.04*^ƚ^
Other/genitourinary infections	0 (0)	2 (1.2)	
ALOS			
Major procedures, days, median (IQR)	5 (4-7)	3 (3-5)	<0.0001**^¶^
Minor procedures, days, median (IQR)	2 (2-2)	1 (1-2)	

**Table 3:** Cost of antibiotics and estimate of reduction AMT: antimicrobial therapy; PKR: Pakistani rupee

Procedure	Total AMT cost (PKR), 2016	Estimated AMT cost (PKR), 2017	Actual AMT cost (PKR), 2017	Cost saved (PKR), 2017	Budget saved on AMT in 2017
Obstetrics					
Vaginal delivery	47,179	51,985	11,248	40,737	78.40%
Cesarean delivery	160,938	224,180	109,738	114,442	51.10%
Total	208,117	276,165	120,986	155,179	56.20%
Gynecology					
Minor procedures	15,825	32,976	6,774	26,202	79.50%
Major procedures	54,108	72,562	53,415	19,147	26.40%
Total	69,933	105,538	60,190	45,348	42.70%

## Discussion

In Pakistan, women belonging to the low and lower middle-classes can only afford to seek healthcare from either government hospitals or those run by charitable trusts. The majority of these hospitals provide free services or only charge nominally. Due to the burden imposed by such patients, most of these facilities have little or no focus on service quality and standard of care. This could be attributed to limited resources, no health insurance provision by the government, and a lack of awareness about quality standards. Some physicians working in these hospitals have fixed mindsets and practices that they are unwilling to change [15]. However, with few exceptions, the situation of healthcare quality is not much different in private hospitals that generate high revenues. To ensure quality care, a lot of effort and commitment is required, which include continuous planning, use of standard management protocols, and training of healthcare personnel, monitoring, and evaluation. This is difficult to achieve in the absence of focused national or institutional quality care policies [3].

Similar challenges were encountered when TIH took over the administration of an existing maternity hospital. The clinicians along with the management of TIH developed a strategy to ensure quality care in accordance with the institutional vision. Guidelines and protocols were developed and implemented stepwise. One of the biggest challenges was to change the mindset and practices of physicians. This change was made possible by the motivation and supervision of the management as well as the determination of the team to achieve quality objectives [16]. The confidence of the physicians was built gradually on the evidence of promising clinical outcomes.

Anemia was identified as one of the major and most modifiable parameters as the correction of anemia well before the expected date of delivery or surgery can reduce morbidity and mortality in obstetric practices. Correction of anemia increases tolerance to hemorrhage, accelerates healing, and decreases the need for blood transfusion, and brings down the risk of infections [17-18]. Awareness was raised to influence patients’ attitudes and behaviors in general and to highlight the importance of hygiene to avoid worm infestation and diarrhea in particular. The patients were made aware of appropriate ways of iron supplementation, cost-effective sources of iron and folic acid like red meat, liver, dates, beans, gourd, etc. Howyida et al. have found significant improvement in overall knowledge-related practices toward healthy nutritional habits after the institution of nutritional educational guideline intervention among Egyptian pregnant women, and, subsequently, 24% decreased prevalence of anemia [19].

Transfusion rate decreased significantly (5.4% vs. 0.6% p<0.001) in obstetrics. Surbek et al. have shown that active intervention early in pregnancy reduces peripartum blood transfusion rates [20]. In gynecologic procedures, data do not show a significant decrease (16.1% vs. 17.9%). This can be explained by the fact that gynecologic procedures more than doubled in 2017 (67 vs. 154), and the nature of procedures had also changed with the inclusion of complicated as well as oncology cases. In such cases, iron deficiency was not the only cause of anemia and, therefore, blood transfusion remains the best option for treatment of anemia and hemorrhage due to the aggressive nature of these diseases and surgical procedures.

Appropriate use of AMT was an important factor of concern. It was observed that a single dose or 24-hour prophylaxis in major surgical procedures was as effective as 7-10 days of AMT in limiting SSI. Similarly, there was no difference in outcomes in women who underwent VD and did not take AMT compared to those who took 7-10 days of AMT. Therefore, to achieve minimization in the risk of infection and enhancement in healing, efforts should be made for improvement in malnourishment, hygiene, unsafe practices, and the treatment of anemia rather than prolonging the use of AMT. Antimicrobial prophylaxis was prescribed either as a single dose or for 24 hours where indicated [6,21]. Monitoring and evaluation of data on regular basis ensured motivation as well as accountability.

Interestingly, with this decrease in the use of AMT, the rate of SSI decreased significantly from 7.1% in 2016 to 3.6% in 2017 among CD procedures, and from 13.5% to 2.8% in gynecologic procedures. Steiner et al. conclude in their review that the implementation of standard protocols including infection control practices and health education in addition to the judicious use of AMT limits SSI in gynecologic procedures [22]. Prolonged AMT compromises immunity and general health and thereby increases the risk of infection and drug resistance [23]. The cost of AMT decreased by 78.4% in VD, 51.1% in CD, 79.5% in minor gynecology procedures, and 26.4% in major procedures. Resources need to be utilized appropriately and with justification at all times. In institutions with resource constraints, this should be an important consideration [24].

ALOS was significantly reduced with the introduction of daycare services and a reduction in the duration of AMT. Pre-admission optimization of health involving outpatient health education of women, including that related to wound care, were contributing factors [5]. Significant work has been done worldwide to decrease ALOS and to ensure early discharges to decrease hospitalization charges and to increase the availability of beds [25].

The key strength of this study is that the data was extracted from the electronic data system [Health Management Information systems (HMIS)]. Another strength is that multiple quality clinical parameters were explored at one point in time, that is, the frequency of anemia and blood transfusion, duration and cost of AMT, and ALOS and SSI. The study is limited by the absence of data from November 2015, the time when services at the hospital were taken over by TIH. HMIS was introduced in January 2016 and complete and authentic data was only available after it was fully implemented. Therefore, the true pre-intervention status could not be assessed as certain changes in practices and subsequent decline in blood transfusions had started to occur much earlier, although standard protocols were only implemented from the third quarter of 2016.

Note: we are maintaining and strengthening our quality standards and are in the process of continuous improvement of clinical outcomes. Currently, as a policy, unless otherwise indicated, we do not prescribe antimicrobials in VD and minor gynecology procedures and give 24-hour antimicrobial prophylaxis for major surgeries. Our indicators for the last quarter of 2019 are summarized in Table 4.

**Table 4:** Clinical quality indicators for the last quarter of 2019 AMT: antimicrobial therapy; SSI: surgical site infections

Indicators	Obstetrics (%)	Gynecology (%)
Pre-delivery anemia	25	-
Blood transfusion rate	2.8	4.6
AMT of >24 hours	6.9	10.3
SSI rate	5.0	1.5

## Conclusions

With adequate commitment and dedication, improvement in clinical practices and outcomes can be achieved in a short period of time, with the implementation of quality standards along with health education among women of low socioeconomic classes.
